# SimCP3—An Advanced Homologue of SimCP2 as a Solution-Processed Small Molecular Host Material for Blue Phosphorescence Organic Light-Emitting Diodes

**DOI:** 10.3390/molecules21101315

**Published:** 2016-09-30

**Authors:** Yi-Ting Lee, Yung-Ting Chang, Cheng-Lung Wu, Jan Golder, Chin-Ti Chen, Chao-Tsen Chen

**Affiliations:** 1 Department of Chemistry, National Taiwan University, Taipei 10617, Taiwan; likewithyou@gmail.com; 2 Institute of Chemistry, Academia Sinica, Taipei 11529, Taiwan; eveningtin@gmail.com (Y.-T.C.); cdstant@yahoo.com.tw (C.-L.W.); 3 Department of Applied Chemistry, National Chiao Tung University, Hsinchu 30050, Taiwan; jangolder@gmx.net

**Keywords:** solution process, molecular host material, high triplet gap energy, SimCP2, blue phosphorescence OLED

## Abstract

We have overcome the synthetic difficulty of 9,9′,9′′,9′′′,9′′′′,9′′′′′-((phenylsilanetriyl)tris(benzene-5,3,1-triyl))hexakis(9H-carbazole) (**SimCP3**) an advanced homologue of previously known **SimCP2** as a solution-processed, high triplet gap energy host material for a blue phosphorescence dopant. A series of organic light-emitting diodes based on blue phosphorescence dopant iridium (III) bis(4,6-difluorophenylpyridinato)picolate, FIrpic, were fabricated and tested to demonstrate the validity of solution-processed **SimCP3** in the device fabrication.

## 1. Introduction

**Scheme 1:**
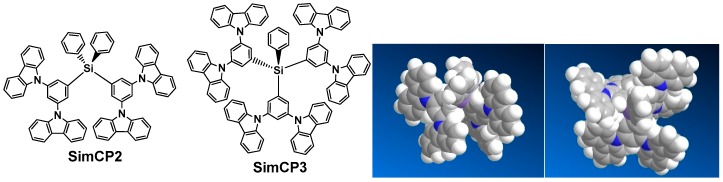
Chemical structure of **SimCP2** and **SimCP3**. MM2 energy minimized structures (by Chem 3D) are shown on the right, respectively.

An increasing number of high-performance blue phosphorescence organic light-emitting diodes (OLEDs) can be found in literature reports. Although remarkable electroluminescence (EL) efficiency as high as ~27%, ~51 lm/W, or ~54 cd/A based on sky-blue phosphorescence dopant iridium (III) bis(4,6-difluorophenylpyridinato)picolate (FIrpic) has been achieved [1,2,3,4,5,6,7], vacuum-thermal-evaporation is a common device fabrication process, but it is not favorable for mass production or large panel fabrication. Accordingly, for more desirable production or fabrication process, there have been a number of solution-processable small molecular host materials for blue phosphorescence organic light-emitting diodes in recent years [8,9,10,11,12,13,14]. We and others have developed and demonstrated bis(3,5-di(9*H*-carbazol-9-yl)phenyl)diphenylsilane (**SimCP2** in Scheme 1) as one of high-performance solution-processable small molecular host materials for yellow, green, and blue phosphorescence OLEDs [15,16,17,18,19,20,21,22]. More recently, **SimCP2** hosted FIrpic OLEDs were reported as having EL efficiency reaching ~11%, ~11 lm/W, or 23 cd/A [23]. Nevertheless, the EL of these FIrpic OLEDs with solution-processed light-emitting layer is far less efficient than those OLEDs fabricated by vacuum-thermal-evaporation. For blue phosphorescence OLEDs, solution-processable small molecular hosts with a high triplet energy gap (*E*_T_) greater than 2.6 eV of the benchmark sky-blue FIrpic are still rare and highly demanded in the field. As our ongoing effort to develop high-performance solution-processable small molecular host materials for blue phosphorescence OLEDs, we have recently overcome synthetic difficulties and successfully prepared 9,9′,9′′,9′′′,9′′′′,9′′′′′-((phenylsilanetriyl)tris(benzene-5,3,1-triyl))hexakis(9*H*-carbazole) (**SimCP3**) (Scheme 1) as an advanced homologue of **SimCP2**. Herein, we report the synthesis and characterization of **SimCP3** in full detail. A series of **SimCP3**-hosted OLEDs are fabricated and tested to demonstrate its viability as a solution-processed host material for sky-blue phosphorescence OLEDs.

## 2. Results and Discussion

**Scheme 2:**
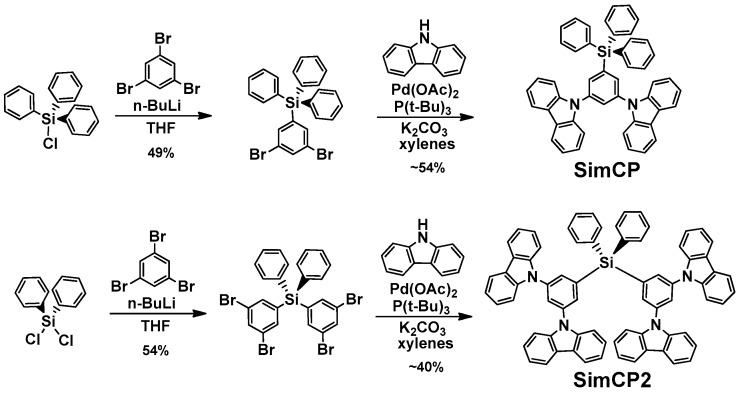
Synthetic preparation of **SimCP** and **SimCP2**.

In our earlier reports [16,24,25], both 9,9′-(5-(triphenylsilyl)-1,3-phenylene)bis(9*H*-carbazole) (**SimCP**) and **SimCP2** were synthesized in a similar method (Scheme 2). Either chlorotripheylsilane or dichlorodiphenylsilane was reacted with monolithiated 1,3,5-tribromobenzene to furnish 1,3-dibromophenyl substituted thriphenylsilane and bis-1,3-dibromophenyl substituted diphenylsilane, respectively. The silane compounds were then brought to a palladium-catalyzed amination reaction with excess amount of carbazole. Both **SimCP** and **SimCP2** were obtained with a reasonable isolation yield of 54% and ~40%, respectively, in the final amination reaction. It is conceivable that the reaction yields will be lower than that of **SimCP2** if the higher homologue **SimCP3** is prepared by a same reaction sequence shown in Scheme 2.

**Scheme 3:**
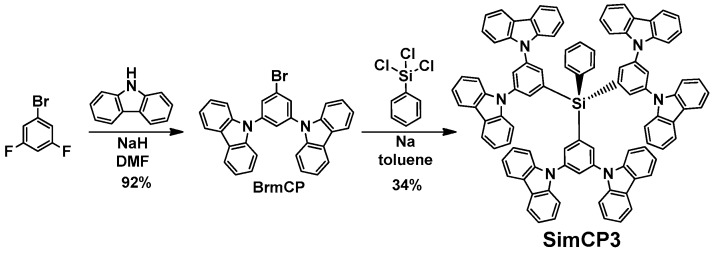
Synthesis of **SimCP3** from BrmCP via Würtz–Fittig reaction.

Due to the much more partial amination products formed in the reaction of synthesizing **SimCP3**, we obtained a series of product mixtures, which were hard to separate by column chromatography. In addition, the purification of **SimCP3** required a tedious process in order to remove the excess amount of carbazole used in the reaction. Alternatively, we have been able to synthesize **SimCP** by using mono-lithiated 9,9′-(5-bromo-1,3-phenylene)bis(9*H*-carbazole) (BrmCP) to react with chlorotripheylsilane. However, the same synthetic approach has been problematic in the preparation of **SimCP2** and **SimCP3**, even though a very recent literature has reported the same synthetic approach in the preparation of **SimCP2** and **SimCP3** [23]. We also tried the approach of Grignard reaction (Mg instead of *n*-butyllithium), but it did not work out well either. It seems to us that either lithiated or magnesiated BrmCP is relatively unstable under the reaction condition. In the case of **SimCP2** and **SimCP3**, the chance of either lithiated or magnesiated BrmCP reacting more than once with dichlorophenylsilane or trichlorophenylsilane is rather low. In addition, the reaction is quite sensitive to the purity of dichlorophenylsilane or trichlorophenylsilane, which is prone to hydrolysis and generates hydrochloric acid that is detrimental to the reaction. Finally, we were able to synthesize and isolate pure **SimCP3** in multi-gram scale via reaction with sodium metal, i.e., a Würtz–Fittig-type coupling reaction between chlorosilane and aryl halide (Scheme 3) [26,27]. Instead of a carbon anion of BrmCP, a more feasible silyl anion of trichlorophenylsilne is involved in the coupling reaction.

To facilitate the **SimCP3** preparation, we improved the synthesis of BrmCP with a significantly better isolation yield of >90% [12,28]. Regarding the Würtz–Fittig-type coupling reaction, **SimCP3** was furnished in only modest yields around 30% due to the competition reaction of the potential silicon–silicon bond formation of oligosilane or polysilane [29,30]. However, the desired product was much easier to isolate from the product mixture. In this reaction, we also observed silanol side products, although they were readily removed by column chromatography. In addition, there was no excess amount of carbazole involved in the reaction; hence, no extra purification process was required for **SimCP3**. These are the main advantages of the Würtz–Fittig reaction over other synthetic methods of preparing **SimCP3**. Several spectroscopic and physical methods were used to characterize the homogeneity of thin film and the necessary optoelectronic properties of **SimCP3** as a solution-processable host material for FIrpic OLEDs.

**Figure 1:**
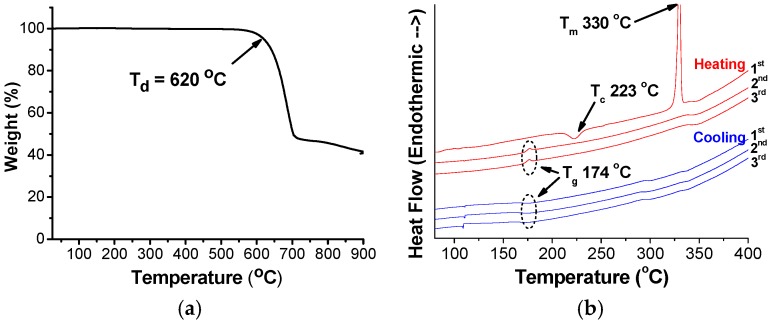
TGA (**a**) and DSC (**b**) thermograms of **SimCP3**.

First, as shown in Figure 1, **SimCP3** is very thermally stable with a thermal decomposition temperature (*T*_d_) as high as 620 °C, where 5% weight loss was determined by thermogravimetric analysis (TGA) under a nitrogen atmosphere. Second, **SimCP3** exhibits amorphous or semi-amorphous characteristic based on the thermograms of differential scanning calorimetry (DSC). As shown in Figure 1, **SimCP3** has melting temperatures (*T*_m_), crystallization temperatures (*T*_c_), and glass transition temperatures (*T*_g_) of 330, 223, and 174 °C, respectively. Two very weak exothermic signals around 290 and 330 °C may be the inhibited crystallization process of thermally annealed **SimCP3**. The *T*_m_ and *T*_c_ of **SimCP3** disappeared, and *T*_g_ was observed after the first heating scan, indicative of the amorphous tendency of **SimCP3**.

**Figure 2:**
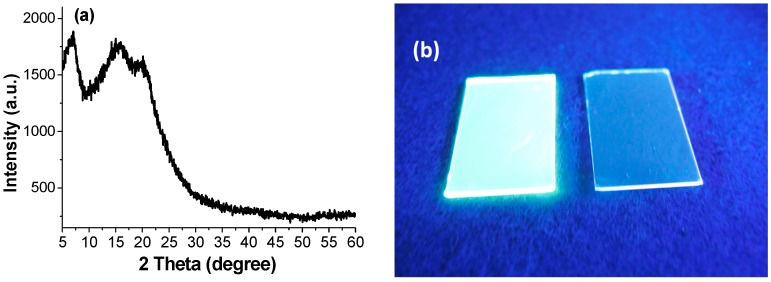
(**a**) XRD spectra of the solution drop-casting thin film of **SimCP3** on silicon wafer; (**b**) PL images of solution spin-coated thin films of **SimCP3**: FIrpic (~10 wt %) and **SimCP3**.

**Figure 3:**
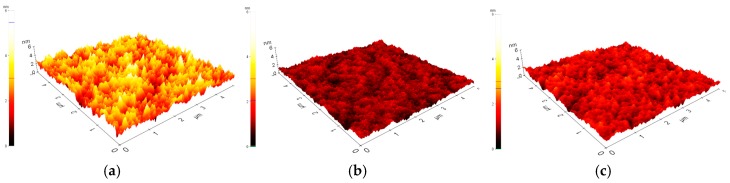
5 × 5 μm AFM images of ITO/PEDOT:PSS (**a**); ITO/PEDOT:PSS/**SimCP3** (**b**); and ITO/PEDOT:PSS/**SimCP3**:FIrpic (10 wt %) (**c**).

Similarly, amorphous or semi-amorphous characteristics are evident from the X-ray diffraction (XRD) spectrum (Figure 2a) of the solution-casting thin film of **SimCP3**. The XRD spectrum is basically a featureless halo, indicative of an amorphous nature. Macroscopically, the homogeneity of the solution spin-coated **SimCP3** thin film is evident from the photoluminescence (PL) images of the neat film and FIrpic-doped **SimiCP3** thin films (Figure 2b), which show uniform color and no sign of agglomeration or phase separation. Microscopically, atomic force microscopy (AFM) demonstrated the homogenous and flat surface morphology of **SimCP3** thin films with or without a FIrpic dopant (Figure 3). Whereas thin film of poly(3,4-ethylenedioxythiophene) doped with poly(styrenesulfonate) (PEDOT:PSS) had a higher surface roughness of 0.68 nm, a rather small values of root-mean-square (RMS) roughness of 0.25 and 0.33 nm was observed for the thin film of **SimCP3** and **SimCP3**:FIrpic (10 wt %), respectively. Regarding the flatness of the thin films, none of the discernable features in AFM surface images had a height over 5.5 nm, 2.1 nm, and 2.7 nm, for PEDOT:PSS, **SimCP3**, and **SimCP3**:Firpic thin films, respectively.

**Figure 4:**
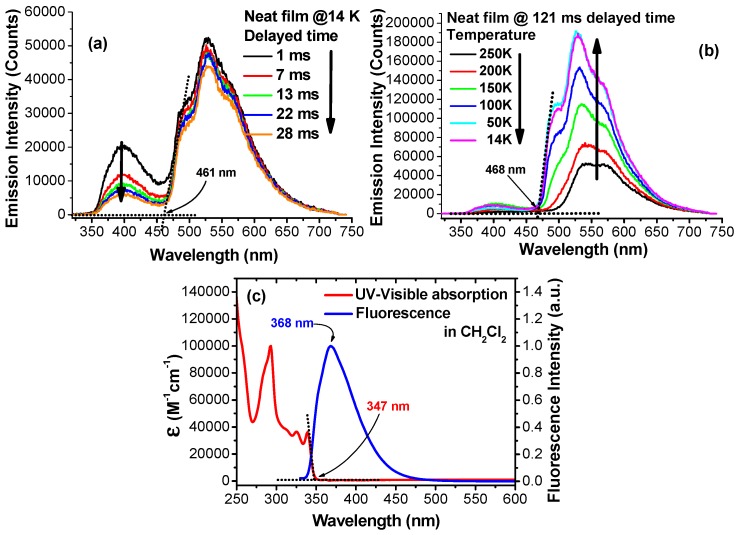
(**a**) Variable time-gated PL spectra of **SimCP3** thin film; (**b**) Variable temperature, 121 msec time-delayed PL spectra of **SimCP3** thin film; (**c**) Room temperature absorption and PL spectra of **SimCP3** in dichloromethane solution.

The most important property of the host material for a blue phosphorescence dopant such as FIrpic is *E*_T_. We estimated the *E*_T_ of **SimCP3** as a neat thin film with time-gated acquisition PL spectra at low temperatures (Figure 4a,b). From spectra with either variable delayed time or variable low temperature, it can be confirmed that the emission band with a λ_max_ around 400 nm is the delayed fluorescence and a λ_max_ around 525 nm is the phosphorescence. By taking the onset wavelength of the high energy edge of phosphorescence bands, we obtain ~461–468 nm (2.69–2.65 eV) as the optical energy gap for *E*_T_. From our measurements, the *E*_T_ of **SimCP3** is sufficiently larger than that of FIrpic, ensuring triplet state exciton on the phosphorescence dopant rather than its host **SimCP3**. The UV-visible absorption and fluorescence spectra of **SimCP3** in solution are illustrated in Figure 4c.

**Figure 5:**
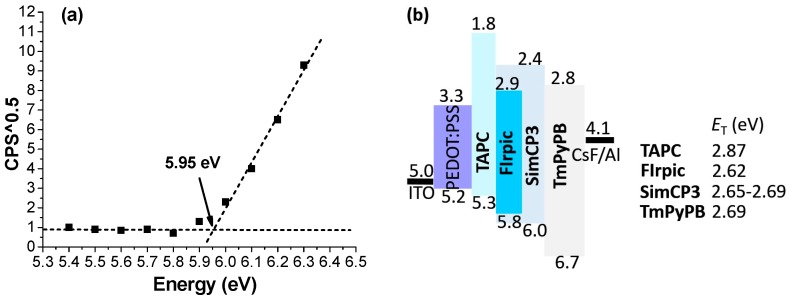
(**a**) Low-energy photoelectron spectra of **SimCP3**; (**b**) Energy level alignment of materials involved in OLEDs.

Knowing an optical energy gap of 3.57 eV (347 nm shown in Figure 4c) and the HOMO energy level of 5.95 eV (Figure 5a), which was acquired by a low-energy photoelectron spectrometer Riken-Keiki AC-2, the LUMO energy level of **SimCP3** can thus be calculated as ~2.4 eV. Together with the HOMO and LUMO energy levels and *E*_T_ of TAPC (hole transporting or electron blocking material) [31,32], FIrpic [32], and TmPyPB (electron transporting or hole blocking material) [33], the energy level alignment of materials involved in OLEDs is depicted in Figure 5b. Therefore, TAPC, SimCP3, and TmPyPB all have HOMO and LUMO energy levels that encompass those of FIrpic, and FIrpic has the smallest *E*_T_ among all materials involved in the OLED fabrication. This is a good set of materials enabling the confinement of triplet exciton on the FIrpic phosphorescence dopant. Accordingly, we fabricated three types of FIrpic OLED having **SimCP3** as a solution-processed host material: a single-layer device (SL)—ITO/PEDOT:PSS/**SimCP3**:FIrpic/CsF/Al—a bilayer device (BL)—ITO/PEDOT:PSS/**SimCP3**:FIrpic/TmPyBP/CsF/Al—and a trilayer device (TL)—ITO/PEDOT:PSS/TAPC/**SimCP3**:FIrpic/TmPyBP/CsF/Al. After optimization, the dopant concentration of FIrpic was 4 wt % in all three devices. Except for TmPyBP and CsF/Al cathode, each thin film layer of such FIrpic OLEDs was fabricated by solution process, i.e., the spin-coating method.

**Figure 6:**
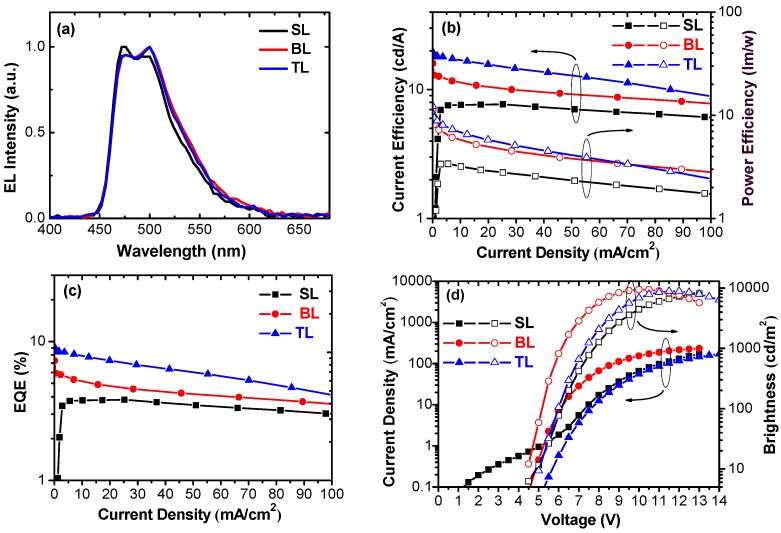
Electroluminescence characteristics of **SimCP3-**hosted FIrpic OLEDs. (**a**) EL spectra at ~10 volt; (**b**) Current density dependent current efficiency and power efficiency; (**c**) Current density dependent external quantum efficiency; (**d**) Voltage dependent current density and brightness.

EL characteristics of SL, BL, and TL devices are summarized in Figure 6 and Table 1. All three devices exhibit typical sky-blue emissions of FIrpic with a λ_max_ of ~468 nm, although a variation of emission shoulder band around 500 nm (Figure 6a) was observed. Since both BL and TL devices have a similar intensity of the shoulder band and are both more intense than that of the SL device, it can be logically inferred that TmPyPB effectively blocks the triplet exciton of FIrpic, confining the charge recombination mostly in the **SimCP3**:FIrpic light-emitting layer (i.e., farther away from the cathode surface) [19]. Therefore, the same reason can be ascribed to the low EL efficiency of the SL device (Figure 6b,c), where the triplet exciton of FIrpic generates in an area too close to the cathode surface and hence is prone to being quenched. Similarly, the TAPC of the TL device plays a role of inhibiting triplet exciton from contacting the anode of the device. Therefore, the EL efficiency of the TL device is promoted further when compared with the BL device. The maximum external quantum efficiency (EQE), current efficiency (CE), and power efficiency (PE) of the TL device are ~9%, ~20 cd/A, and 12 lm/W, respectively. However, the TL device has the lowest current density and the highest turn-on voltage—around 5 V at 1 cd/m^2^ (see Figure 6d)—among the three devices. This can be attributed to the two exciton blocking layers in the device, which limits the current density and elevates the driving voltage of the device. Nevertheless, all three devices exhibited relatively mild efficiency roll-off. Even at the high brightness of 3000 cd/m^2^ (about 38, 30, and 19 mA/cm^2^ of the SL, BL, and TL devices), an EQE of 3.6%, 4.5%, and 7.4% were achieved by the SL, BL, and TL devices, which are 95%, 62%, and 81% of the maximum EL efficiency, respectively (Figure 6c). Compared with those of vacuum-thermal-evaporation OLEDs [2,4], the efficiency roll-off observed for the three devices is relatively modest. 

## 3. Materials and Methods

### 3.1. General Instrumentation

^1^H- and ^13^C-NMR spectra including distortionless enhancement by polarization (DEPT) were measured on a Bruker AV-400 MHz or AV-500 MHz NMR Fourier transform spectrometer (Bruker, Billerica, MA, USA) at room temperature. Elemental analyses (on a FLASHEA 1112 Series, Thermo Electron Corporation, Waltham, MA, USA), matrix-assisted laser desorption/ionization time-of-flight mass spectroscopy (MALDI-TOF MS, The New Ultraflextreme^TM^, Bremen, Germany), and fast atom bombardment (FAB) mass spectroscopy (FAB-MS, JMS-700 double focusing mass spectrometer, JEOL, Tokyo, Japan) were performed by the Elemental Analyses Laboratory and Mass Spectroscopic Laboratory, respectively, in-house service of the Institute of Chemistry, Academic Sinica. Infrared absorption spectra were recorded on a Varian 640-IR FT-IR spectrometer (Palo Alto, CA, USA). The sample of **SimCP3** was ground together with KBr powder and compressed into a thin disc. Thermal decomposition temperatures (*T*_d_′s) of the host materials were measured by thermogravimetric analysis (TGA) using Perkin-Elmer TGA-7 analyzer systems (Waltham, MA, USA). Melting temperatures (*T*_m_′s), crystallization temperatures (*T*_c_′s), and glass transition temperatures (*T*_g_′s) of the host materials were measured by differential scanning calorimetry (DSC) using Perkin-Elmer DSC-6 analyzer systems (Waltham, MA, USA), using a scan rate of 10 °C/min. The X-ray diffraction measurement of the solution-casting thin film was carried out by using a Bruker D8 Advance diffractometer (Billerica, MA, USA) equipped with a Lynxeye detector. The radiation used was a monochromatic Cu Kα bea`m of wavelength λ = 0.154 nm. AFM measurements were carried out with XE-100 from Park Systems (Suwon, South Korea) using a non-contact mode to obtain topography images of the thin films, which were prepared by a spin-coating chlorobenzene solution of **SimCP3** or **SimCP3**:FIrpic on a PEDOT:PSS-coated ITO-glass substrate. UV-visible absorption spectra were recorded on a Hewlett-Packard 8453 diode array spectrophotometer (Palo Alto, CA, USA). Room temperature fluorescence spectra were recorded on a Hitachi fluorescence spectrophotometer F-4500 (Tokyo, Japan). The ionization potentials (or HOMO energy levels) of **SimCP3** were determined with a low energy photo-electron spectrometer (Riken-Keiki AC-2, Riken Keiki Co., Ltd., Tokyo, Japan). LUMO energy levels were estimated by subtracting the optical energy gap (*E*_g_) from HOMO energy levels. *E*_g_ was determined by the on-set absorption energy from the absorption spectra of the materials as thin film. To measure the triplet energy gap (*E*_T_) of a thin film sample, we established a system with a temperature control of Model 350 (LakeShore Company, Westerville, OH, USA) as low as 10 K by Model 22C/350C Cryodyne Refrigerators (Janis Research Company, Woburn, MA, USA) and a tunable laser with excitation wavelengths of 213, 266, 355, 532, and 1064 nm (Brilliant B laser, Quantel Company, Newbury, Berkshire, UK), which was coupled with a time-delay controller of LP920 flash photolysis spectrometer (Edinburgh Instrument, Kirkton Campus, Livingston, England). We also acquired the triplet energy gap of **SimCP3** in frozen 2-Me-THF by using Fluorolog III photoluminescence spectrometer (Kirkton Campus, Livingston, UK), which was equipped a xenon lamp as excitation light source.

### 3.2. Device Fabrication and Performance Measurements

The ITO substrate was purchased from Buwon Precision Sciences (Taoyuan, Taiwan with a sheet resistance around 25 Ω/sq and a thickness of 100 nm. The hole transport layer—PEDOT:PSS (CH8000)—was purchased from Sigma-Aldrich (St. Louis, MO, USA). The blue phosphorescence dopant FIrpic, the electron transport layer of TmPyPB, and the hole transport layer of TAPC were obtained commercially from Lumtec (Hsinchu, Taiwan). The indium tin oxide (ITO) substrates were pre-coated with the PEDOT:PSS hole transporting layer (HTL) and baked in the air at 150 °C for 10 min. Blends of **SimCP3** and FIrpic in chlorobenzene were spin-coated on ITO/PEDOT:PSS. The active layer was then annealed at 90 °C for 30 min. After spin-coating the active single-layer, the TmPyPB (50 nm), an ultrathin CsF (2 nm) interfacial layer, and then an aluminum cathode (100 nm) were vacuum-thermal deposited. In the triple-layer device, a thin layer (~20 nm) of TAPC was spin-coated first on PEDOT:PSS. In this case, TAPC layer was thermally annealed at 90 °C for 10 min before spin-coating **SimCP3**:FIrpic. A surface profiler (Dektak 150, Veeco Instruments, Plainview, NY, USA) was used for calibrating the thickness of HTL and the active layer. After the deposition of the cathode, the devices were hermetically sealed with glass and UV-cured resins in a glove box (O_2_ and H_2_O concentration below 0.1 ppm). The device’s active area was 0.04 cm^2^ and was defined by the self-made shadow mask applied in the cathode deposition. Current density and voltage characteristics were measured by a dc current–voltage source meter (Keithley 2400, Tektronix, Beaverton, OR, USA), and the device brightness (or electroluminance, cd/m^2^) and EL spectra were monitored and recorded with a spectrophotometer (PR650; Photo Research, Syracuse, NY, USA). The EQE of OLEDs was calculated from the luminance, current density, and the EL spectra, assuming the EL of the OLED is isotropic, i.e., a Lambertian emission [34].

### 3.3. Materials Preparation

All chemical and reagents were obtained from commercial suppliers and used without further purification. Solvents were purified according to the standard procedures. Unless specified condition, all reaction were performed under a nitrogen atmosphere using standard Schlenk techniques. Materials involved in OLED fabrication, 4,4′-(cyclohexane-1,1-diyl)bis(*N*,*N*-di-*p*-tolylaniline) (TAPC), and 3,3′-(5′-(3-(pyridin-3-yl)phenyl)-[1,1′:3′,1′′-terphenyl]-3,3′′-diyl)dipyridine (TmPyPB) were purchased from Lumtec (Hsinchu, Taiwan) and Ultra Fine Chemical Technology Corp. (Degussa, Dusseldorf, Germany), respectively. They were used as received without further purification.

Synthesis and characterization of *9,9′-(5-Bromo-1,3-phenylene)bis(9H-carbazole)*, BrmCP. The synthesis and isolation of BrmCP are modified from a known procedure of a patent report [28]. To an anhydrous DMF solution (200 mL) containing sodium hydride (60% suspension in oil, 13 g, 0.31 mole), carbazole (52 g, 0.31 mol) was slowly added. The mixture was stirred for 1 h at room temperature. After cooling in an ice bath, 3,5-difluorobromobenzene (12 mL, 0.1 mol) was added dropwise. The solution mixture was then heated at 130 °C for 12 h. After cooling, an excess amount of ethanol–water mixture (10:1) was added and stirred, resulting in white precipitates. The product was isolated by suction filtration, and further purification was achieved by zone-temperature sublimation to afford white solid (44.7 g, 92%). ^1^H-NMR (400 MHz, CDCl_3_): δ (ppm) 8.13 (d, 4H, *J* = 7.6 Hz), 7.85 (s, 2H), 7.78 (s, 1H), 7.53 (d, 4H, *J* = 7.6 Hz), 7.45 (t, 4H, *J* = 7.6 Hz), 7.32 (t, 4H, *J* = 7.6 Hz). ^13^C-NMR (100 MHz, CDCl_3_): δ (ppm) 140.72, 140.55, 128.89, 126.58, 124.32, 124.18, 124.04, 120.98, 120.77, 109.81. MALDI-HRMS: calcd MW 486.0726, *m*/*z* = 486.0705 (M^+^).

Synthesis and characterization of *9,9′,9′′,9′′′,9′′′′,9′′′′′-((phenylsilanetriyl)tris(benzene-5,1,3-triyl))hexakis(9H-carbazole)* (**SimCP3**). To a refluxing toluene (4 mL), sodium metal (0.2 g, 8.3 mmol) was added. The mixture was stirred for 10 min. Then, BrmCP (2.0 g, 4.0 mmol) and trichloro(phenyl)silane (0.2 mL, 1.3 mmol) dissolved in toluene (4 mL) were added slowly to the refluxing toluene containing sodium metal through an additional funnel. After 3 h of reaction, the reaction solution was cooled to room temperature and an excess amount of methanol was added. The resulting precipitations were isolated by suction filtration and subjected to column chromatography (silica gel, dichloromethane/hexanes: 2/3). A white solid was obtained with a yield of 34% (0.6 g). FT-IR (λ, cm^−1^): 3044 (*w*), 3013 (*w*), 1623 (*w*), 1580 (*s*), 1490 (*m*), 1480 (*m*), 1447(*s*), 1416 (*m*), 1374 (*m*), 1330 (*s*), 1309 (*s*), 1227 (*s*), 1191 (*m*), 1155 (*m*), 1120 (*m*), 1098 (*w*), 1027 (*w*), 1002 (*w*), 959 (*w*), 923 (*w*), 904 (*w*), 882 (*w*), 840 (*w*), 798 (*w*), 778 (*w*), 746 (*s*), 706 (*s*), 722 (*s*). UV-Vis (CH_2_Cl_2_): λ_max_ 293, 311, 325, 339 (3.61 × 10^4^ cm^−1^·M^−1^). ^1^H-NMR (400 MHz, CDCl_3_): δ (ppm) 8.07–8.03 (m, 18H), 7.87 (m, 5H), 7.48–7.47 (m, 3H), 7.27 (d, 12H, *J* = 8.4 Hz), 7.15 (t, 12H, *J* = 7.6 Hz), 7.00 (t, 12H, *J* = 7.6 Hz). ^13^C- and DEPT NMR (125 MHz, CDCl_3_): δ (ppm) 140.54, 140.04, 137.07, 136.27 (DEPT-90), 133.69, 132.92 (DEPT-90), 131.40 (DEPT-90), 129.19 (DEPT-90), 127.27 (DEPT-90), 126.47 (DEPT-90), 123.89, 120.74 (DEPT-90), 120.62 (DEPT-90), 109.62 (DEPT-90). MALDI-HRMS: calcd MW 1326.4800, *m*/*z* = 1326.4757 (M^+^). Anal. Found (calcd) for C_96_H_62_N_6_Si: C 86.66 (86.85), H 4.92 (4.71), N 6.37 (6.33).

## 4. Conclusions

In summary, we have overcome the synthetic difficulty of **SimCP3**, an advanced homologue of previously known **SimCP2**. We have examined the amorphous nature and the spectroscopic properties (HOMO and LUMO energy levels as well as the triplet energy gap) of **SimCP3**. Three sky-blue phosphorescence OLEDs have been fabricated with a **SimCP3**:FIrpic light-emitting layer, which was spin-coated from solution. A trilayer device exhibits the highest EL efficiency of ~9%, ~20 cd/A, or ~12 lm/W, demonstrating the viability of **SimCP3** as a solution-processed host material for sky-blue phosphorescence OLEDs.

## Figures and Tables

**Table 1:** Electroluminescence characteristics of **SimCP3-**hosted FIrpic OLEDs.

Device	λ_max_ (nm)	V_on_^a^ (V)	L_max_^b^ (cd/m^2^)	CE ^c^ (cd/A)	PE ^d^ (lm/W)	EQE ^e^ (%)	CIE_x,y_^f^
SL	472, 492	5.0	8078	7.7, 7.0, 7.7	3.4, 3.4, 3.0	3.8, 3.4, 3.8	0.15, 0.32
BL	476, 500	4.5	9440	16.0, 12.7, 10.8	11.1, 7.2, 5.2	7.3, 5.8, 4.9	0.16, 0.34
TL	476, 500	5.0	8880	19.6, 18.0, 17.4	12.3, 9.5, 7.3	9.1, 8.4, 8.1	0.16, 0.34

^a^ Turn-on voltage of 10 cd/m^2^ (V_on_). ^b^ Maximum luminance. ^c^ Current efficiency: maximum, at 100, and 1000 cd/m^2^, respectively. ^d^ Power efficiency: maximum, at 100, and 1000 cd/m^2^, respectively. ^e^ External quantum efficiency: maximum, at 100, and 1000 cd/m^2^, respectively. ^f^ Measured at ~10 volt.
